# Musical Hallucinations in Chronic Pain: The Anterior Cingulate Cortex Regulates Internally Generated Percepts

**DOI:** 10.3389/fneur.2021.669172

**Published:** 2021-05-04

**Authors:** Ashlyn Schmitgen, Jeremy Saal, Narayan Sankaran, Maansi Desai, Isabella Joseph, Philip Starr, Edward F. Chang, Prasad Shirvalkar

**Affiliations:** ^1^Division of Pain Medicine, Department of Anesthesiology and Perioperative Care, University of California, San Francisco, San Francisco, CA, United States; ^2^UCSF Weill Institute for Neurosciences, San Francisco, CA, United States; ^3^Department of Neurological Surgery, University of California, San Francisco, San Francisco, CA, United States; ^4^Department of Speech, Language, and Hearing Science, University of Texas at Austin, Austin, TX, United States; ^5^Department of Physiology, University of California, San Francisco, San Francisco, CA, United States; ^6^Department of Neurology, University of California, San Francisco, San Francisco, CA, United States

**Keywords:** anterior cingulate, chronic pain, perception, deep brain stimulation, musical hallucination

## Abstract

The anterior cingulate cortex (ACC) has been extensively implicated in the functional brain network underlying chronic pain. Electrical stimulation of the ACC has been proposed as a therapy for refractory chronic pain, although, mechanisms of therapeutic action are still unclear. As stimulation of the ACC has been reported to produce many different behavioral and perceptual responses, this region likely plays a varied role in sensory and emotional integration as well as modulating internally generated perceptual states. In this case series, we report the emergence of subjective musical hallucinations (MH) after electrical stimulation of the ACC in two patients with refractory chronic pain. In an N-of-1 analysis from one patient, we identified neural activity (local field potentials) that distinguish MH from both the non-MH condition and during a task involving music listening. Music hallucinations were associated with reduced alpha band activity and increased gamma band activity in the ACC. Listening to similar music was associated with different changes in ACC alpha and gamma power, extending prior results that internally generated perceptual phenomena are supported by circuits in the ACC. We discuss these findings in the context of phantom perceptual phenomena and posit a framework whereby chronic pain may be interpreted as a persistent internally generated percept.

## Introduction

Chronic pain involves the persistence of pain beyond 3 months, beyond the time required for usual tissue healing after injury (1). Moving beyond historical concepts framing pain primarily in terms of nociception, it is now appreciated that chronic pain is associated with the integration of at least three dimensions including somatosensory, affective, and cognitive (2). A central goal of chronic pain research has been to characterize the functional brain network underlying the subjective pain experience in a manner that corresponds to these dimensions (3, 4). Converging evidence from neuroimaging and physiology studies has implicated a central role for brain circuits in the anterior cingulate cortex (ACC) in the affective dimension of both acute and chronic pain states (5–13). Chronic pain also is intricately tied to attention. As part of the salience network, the ACC is functionally connected to the anterior insula and largely responsible for facilitating access to attention of salient stimuli, including motor, motivational, and mood response sensory inputs. Thus, as a potential nexus of affective-emotional networks, the ACC has been studied as a key target for treating chronic neuropathic pain. Though, therapeutic results vary across studies (14–16), the ACC has been specifically proposed as a target for brain stimulation.

Deep brain stimulation (DBS) has been studied and used off-label in the U.S. for intractable neuropathic pain since the 1950s. While initial brain targets included the ventral posterior lateral and medial thalamus, the periaqueductal and periventricular gray (PAG/PVG), and internal capsule, among others, the anterior cingulate cortex is gaining interest (15). In contrast to the somatosensory effects (e.g., paresthesias) produced with ventral posterior thalamus stimulation, stimulation of the ACC has been described in affective terms as reducing the “unpleasantness” or “bothersomeness” of chronic pain (15). Similarly, frontal cingulotomy, a surgical destruction of the frontal cingulum fasciculus previously used to treat intractable chronic pain (17), has produced similar effects. While cingulotomy was reported to not alter patients' perception of pain intensity, it often results in affective changes such that pain was no longer distressing or bothersome (17). More recently, in mouse models, lesions to the ACC have resulted in reduced anxiodepressive behaviors related to pain (18) or a decline in focused attention during cognitive tasks (19). The cases we report below are part of an ongoing clinical trial aimed at elucidating the role of the ACC as a therapeutic target for brain stimulation for chronic pain.

Using intracortical electrical stimulation, a recent study exhaustively synthesized the effects of stimulating 1,789 locations spanning the anterior, mid, and posterior cingulate cortex across 329 patients with drug-resistant epilepsy (20). Behavioral and subjective responses encompassed (anterior to posterior) emotional and interoceptive sensations, complex goal-oriented motor behaviors, somatosensory, and vestibular sensations and visual hallucinations. Temporary stimulation of the ACC has been well-established to produce affective responses, including regulation of anxiety and depression, social distress, and burst laughter with and without mirth (21, 22). The dorsal ACC has further been found to have a role in producing auditory and musical hallucinations in patients with schizophrenia (23). The pathophysiology underlying schizophrenia has been associated with both hypo- and hyperactivation of the ACC during mnemonic and executive function tasks (24). This suggests a critical role of the ACC in both idiopathic and disease-related hallucinations.

Auditory or musical hallucinations have been associated with different neuropsychological disorders (25) such as schizophrenia (26), depression (27), epilepsy (28), hearing loss (25, 29), traumatic brain injury, stroke, and Alzheimer's disease (27). Although, injury to disparate brain structures may result in auditory hallucinations, ACC activity has been proposed to underlie musical hallucinations in experimental settings. Neuroimaging studies have consistently found recruitment of the ACC during music-listening (30), particularly following moments of aesthetic judgement and self-referential appraisal (31, 32). In a meta-analysis of research into the neural correlates of musical emotion, activation of the ACC was reported in 45 and 41% of studies examining *perceived* and *induced* emotions, respectively (33). Evidence also exists that the ACC may be involved in more active musical processes outside of a listening context; Berkowitz and Ansari (34) reported increased ACC activity during melodic improvisation in trained pianists, while Farrugia et al. (35) found that the frequency of occurrence of “earworms,” a type of involuntary musical imagery (further defined in section A Continuum from Involuntary Musical Imagery to Musical Hallucinations) was correlated with cortical thickness in several regions, including the ACC. More directly, spontaneous coactivation of ACC and auditory cortex have been described during simple auditory tasks (36) and auditory imagery during silence (37), in a manner where ACC may be driving gamma band activity in auditory cortex. Together, these data suggest that ACC may exert top-down control over remote cortical regions to modulate the salience of internally generated perceptions, such as musical hallucinations or chronic pain.

Here, we provide a narrative description of two patients with neuropathic pain that experienced spontaneous induction of musical hallucinations (MH) after electrical stimulation of the rostral ACC. These subjects include Patient 1 (P1), a 60-year old woman with a history of phantom limb pain below the right knee, and Patient 2 (P2), a 56-year old man with a history of thalamic stroke. While musical hallucinations were present in some form for P1 prior to a “reawakening” associated with recent right ACC stimulation, P2 experienced musical hallucinations for the first time in his life. By assessing the neural activity of one patient in baseline and post-Right ACC stimulation activity, and comparing the background and experiences of both patients, we sought to better understand the etiology and neural correlates of musical hallucinations in chronic pain.

## Materials and Methods

### Clinical Trial Overview and Brain Surgery

This study contains a subset of findings from an ongoing clinical trial aimed at developing adaptive DBS for chronic pain. As data collection is ongoing, we do not report outcome measures or additional details related to treatment of chronic pain beyond the scope of this article.

To gather details on these subjective experiences, we collected in-person narrative reviews from the patients using video recordings, phone interviews, and quantitative surveys.

Patients were screened for inclusion (e.g., refractory chronic pain for >1 year) and exclusion criteria (e.g., history of untreated psychiatric disorder, cognitive deficits; full inclusion/exclusion criteria can be found at clinicaltrials.gov under NCT03029884). Baseline structural neuroimaging was performed using magnetic resonance imaging (MRI) protocol using thin slice T1 and T2 images for preoperative planning. Each patient was then implanted with the investigational Medtronic Activa PC+ DBS system using frame-based stereotaxy under general anesthesia, through a research agreement between Medtronic Inc and the University of California, San Francisco (UCSF). The Activa PC + S is a bidirectional neural implant that allows simultaneous neural recording of local field potential (LFP) activity and electrical stimulation through the same leads. Neural trajectories were optimized for the anterior cingulate (Medtronic lead 3,387) and orbitofrontal cortices in both hemispheres using BrainLab and Medtronic Stealth software. The anterior cingulate was targeted as per prior reports (38). Briefly, we located the ventral most aspect of the cingulate gyrus on each side measuring 25 mm posterior to the anterior border of the frontal horn of the lateral ventricles on pre-operative MRI T1-weighted scans. The electrode tips were targeted such that the inferior most contact just abutted the corpus callosum.

Patients were allowed to post-operatively recover for 10 days, after which we began recording neural activity from two channels in the absence of stimulation for up to 3 months during which we collected “baseline” neural recordings.

### Stimulation and Neural Local Field Potential Recording

Following initial in-clinic safety testing, each patient's DBS device was programmed weekly with various program sets of stimulation to systematically assess response across brain regions and parameters (Table 1). Stimulation parameters were programmed based on efficacious settings described in DBS chronic pain literature (39, 40).

**Table 1:** Stimulation sets (1–13) programmed onto the patient's implanted pulse generator.

**Set**	**Side**	**Region**	**Left ACC Amp (mA)**	**Left OFC Amp (mA)**	**Right ACC Amp (mA)**	**Right OFC Amp (mA)**	**PW (μs)**	**Fq (Hz)**
1	Bilateral	ACC	2		2		450	140
2	Sham	Sham	0	0	0	0	0	0
3	Bilateral	OFC	4		4		210	140
4	Bilateral	ACC + OFC	2	4	2	4	450/210	140
5	Bilateral	ACC	5.2		4.6		450	140
6	Bilateral	ACC + OFC	3.5	4.5	4	4.5	450/100	140
7	Left	ACC + OFC	4	4.5			450/100	140
8	Left	OFC	5				210	100
9	Left	ACC	6				450	40
10	Left	ACC	6		5.6		450	100
11	Sham	Off	0	0	0	0	0	0
12	Left	OFC		5			450	100
13^*^	**Right**	**ACC**			**6**		**450**	**40**

* *The patient did not report any musical hallucinations or other psychiatric or behavioral changes prior to Set 13, during which the patient reported initiation of musical hallucinations for the first time in over 3 years. The bold values highlight the stimulation parameters at the time of musical hallucination onset*.

*Sets 1–13 ~5 months of programmed DBS (at 5 days to 2 weeks per set)*.

We collected at-home LFP recordings following the initial Right ACC stimulation program that was associated with MH (Table 1, Set 13), during which various stimulation parameters, including sham stimulation were delivered. LFP data were only analyzed from pre-stimulation (pre-MH) and sham stimulation (post-MH) conditions to minimize stimulation-related artifact. These analyzed recordings were collected over the course of 5 months following MH onset. Patient neural data was captured with a sampling rate of 422 Hz in 30–60 s recording segments manually triggered by the patient using a controller in communication with the implanted neurostimulator. The time-frequency domain data was then downloaded from the patient tablet and uploaded to the research team database for analysis using MATLAB 2019a (Natick, MA). The organized data was then preprocessed using a 1 Hz high pass filter and notch filter at outlier frequency bands.

### Music Listening Task

During initial in-clinic testing prior to the initiation of brain stimulation, study participants engage in acute cognitive and behavioral tasks for baseline measurements. Included amongst these tasks performed during LFP recording was a structured music listening activity, in which the patient was instructed to sit quietly and listen to four alternating positive and negative valence classical pieces: Die Post and Der Leiermann composed by Franz Schubert and Chopin C Major and C Minor composed by Frédéric Chopin. The positive and negative valences were chosen based on tempo, pitch, harmonies, timbre, consonance/dissonance, modality, and physiology. Pain intensity, mood, and pleasure visual analog scores were gathered at the start and 30 s into each piece.

### LFP Preprocessing and Spectral Analysis

Recordings from right ACC were assessed for changes in spectral power during baseline, continuous MH (sham-stimulation), and music listening (ML) recordings. LFPs were divided into overlapping segments across the total length of samples and converted to frequency domain using Welch's Fourier transform method (Pspectrum function) (41). FFT used a Kaiser window, with resolution of 128–256 samples (in a recording segment containing 4,096 total samples) over a frequency range of 1–100 Hz. Through visual inspection, we determined that many recordings exhibited noise at frequencies > 45 Hz, so only data below this frequency were processed further (1–45 Hz). Raw spectral power for each frequency was then averaged across the recordings and plotted with the standard error of the mean. Empirically determined bands of interest were then box plotted with mean, standard deviation, and individual raw power data for alpha, defined as 8–10 Hz, and low gamma, defined at 35–45 Hz.

The difference in raw power was calculated by subtracting raw power of the averaged baseline (*n* = 8 recordings) from the averaged MH (*n* = 8 recordings) across all frequencies.

### Statistics

Multiple paired *t*-tests were performed for comparison of alpha (8–10 Hz) and low gamma (35–45 Hz) in the baseline (pre-MH), MH (post-MH onset), and music listening (ML) conditions. Results were confirmed with a one-way ANOVA comparing baseline, MH, and ML datasets for alpha (*p* < 0.01) and low gamma (*p* < 0.01). Correction for multiple comparisons used FDR correction with Benjamini-Hochberg method (42).

### Lead Localization and Electrical Volume of Tissue Activation

Lead-DBS, an open-source MATLAB package, was used to localize the DBS electrodes and calculate the volume of tissue activated (VTA) (43). The post-operative computed tomography (CT) scan was registered to the pre-operative T1-weighted MRI using the Advanced Normalization Tools (ANTs) toolkit to perform a two-stage linear registration (44). Imaging was then normalized into MNI 1522009b space (45). Normalization was achieved using a five-stage approach within the ANTs toolkit. First, two linear registration steps were taken followed by a whole-brain symmetric image normalization (SyN) registration stage. Finally, the images were transformed using two non-linear SyN-registration stages focusing on subcortical refinement. To correct for brainshift, a refined affine transform was performed using the brainshift-correction module in Lead-DBS. DBS electrodes were then manually localized.

The Brainnetome atlas was used for 3D visualization and calculating the VTA overlap. VTA was estimated using finite element models [see (46)]. Conductivity values for white and grey matter were set to 0.14 and 0.33 S/m, respectively. A value of 0.2 V/mm was used as a threshold to determine the VTA from the potential gradient.

## Results

### ACC Stimulation Induced Musical Hallucinations in Two Patients

The two patients described in this review both have a rich history in musical performance and a complex history with chronic neuropathic pain. Two other patients (neither with musical training) in the parent clinical trial denied any musical hallucinations after ACC stimulation.

#### Narrative Description of Patient 1

P1 is a 60-year old woman with a history of refractory phantom limb pain below the right knee.

**Figure 1:**
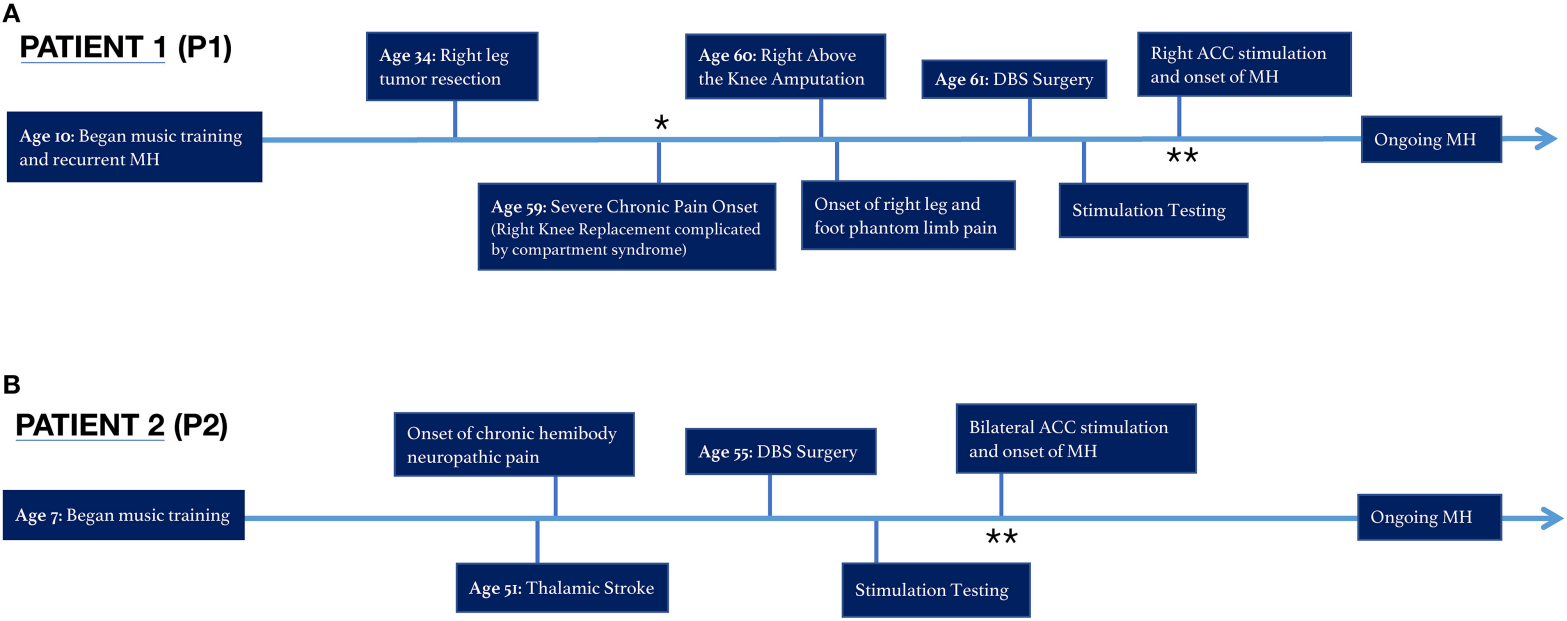
Timeline of chronic pain and musical hallucinations for Patients 1 and 2. **(A)** Patient 1 (P1) experienced continuous MH throughout her life, until the onset of severe chronic pain at age 59. The MH became less frequent and noticeable until the patient received brain stimulation to the right ACC. **(B)** Patient 2 (P2) developed MH for the first time during bilateral ACC stimulation. *MH muted, **MH onset.

Approximately 25 years prior to severe chronic pain onset, the patient was diagnosed with a non-metastatic desmoid tumor in the right lower leg, which was resected successfully but left the patient with compromised mobility and mild residual pain, rated 2–3 on a 0–10 numerical rating scale (NRS). The patient underwent several minimally successful arthroscopies in the decades following, before undergoing a full knee replacement 24 years after initial resection. The knee replacement surgery was complicated by compartment syndrome, requiring fasciotomy and debridement, and subsequently resulted in residual constant pain uncontrolled by medications in the inpatient or outpatient setting. One year later, an above the knee amputation of the right leg was performed to improve pain, and subsequently shifted the patient's reported pain down from a 9–10 to a 6–7 on the 0–10 scale. However, the patient's residual phantom limb pain, described as a constant sharp, shooting, burning and squeezing pain in the phantom leg and foot, has persisted despite treatment attempts with several classes of opioid and nerve pain medications, transcutaneous electrical nerve stimulation (TENS), two trials of spinal cord stimulation, lidocaine injections to the femoral, and sciatic nerves and the stump, biofeedback, physical therapy, hyperbaric oxygen therapy, and alternative treatments, such as acupuncture, medical marijuana, meditation, and off-label medications. The patient was enrolled into a DBS trial with chronically implanted leads in the bilateral ACC and orbitofrontal cortex (OFC) to evaluate the potential benefit of an experimental therapy as outlined in Figure 1.

P1 has reported a longstanding history of musical hallucinations predating the onset of neuropathic pain and delivery of deep brain stimulation. P1 began taking violin lessons at age 10 and continued performing while studying violin performance at the undergraduate and doctorate level. During this time, the patient would perceive familiar and unbothersome persistent involuntary classic musical imagery in the absence of an external source. This perception of music when none is externally playing, a conventional definition of musical hallucinations (47), had been an active and routine phenomenon throughout the patient's life. However, when the patient developed chronic right lower extremity pain in 2016 followed by refractory phantom limb pain in 2017, the hallucinations became “muted.” In the presence of severe chronic pain, the patient's hallucinatory activity had dimmed below any noticeable perceptual threshold. Of note, the patient lacks common underlying risk factors for musical hallucinations such as hearing loss, psychosis, schizophrenia, bipolar disorder, obsessive compulsive disorder, temporal lobe epilepsy, or other central nervous system pathology, nor was she taking any pertinent medications (48, 49).

This muted state of prior MH persisted even after implant of the DBS device, and throughout stimulation of left, right, bilateral OFC, and left and bilateral ACC, including “safety testing” of these sites at varying parameters in 3–5 min sessions. However, when we commenced week-long continuous stimulation of right ACC, the patient reported the reemergence of a “strong, internal music,” experienced for the first time in over 3 years. The newly discovered salience of MH was reported approximately 24 h after commencing right ACC stimulation. Upon reflecting on the newfound MH, the patient stated, “stimulation allowed that part of my brain to awaken.” She interpreted that until that point, “the pain had overwhelmed and taken over that part of my brain.” These musical hallucinations, noted by the research team at the time of onset below, have persisted throughout each day continuously in the background since this time, nearly identical as described prior to her pain onset. Since reemerging, MH have continued unabated during periods of no stimulation, sham stimulation, and subsequent stimulation programs. She can usually identify the specific song being heard, the key of the song, as well as the particular recording or version of the performance she is hearing. Of note, she is not bothered by these MH nor do they interfere with daily functioning.

During this onset, the patient reported vividly “hearing” Beethoven's seventh sonata in a repeated pattern in which she was able to transpose it, adjust the tempo, and overlay the notes onto other sequences. She had noted that this while this was not unfamiliar, it was loud and frequent for the first time since the onset of chronic pain 3 years prior. At this time, the patient reported a dissociation between perceived pain intensity and attention to pain. This activity persisted continuously when stimulation was off and has been ongoing since this event.

Stimulation parameters that were first associated with reemergence of MH were: Right ACC stimulation, 0–3+ (0 is the most ventral contact), amplitude of 6 mA, pulse width of 450 us, and frequency of 40 Hz, cycling on for 10 min, and off for 5 min (cycling was programmed to preserve battery power). Metrics for comparison of recordings preceding and following this onset, including time of day, subjective pain intensity, and mood were considered (Table 2). Though, the patient has qualitatively described the perceptual change in experienced pain, her pain remains high, and is described as a “vice-like squeezing and burning” with occasional intermittent jolts of sudden unpredictable electrical pain to the right calf, ankle and foot. She also has rare “muscle spasms” in territory of the amputated lower leg.

**Table 2:** Matched sham stimulation recordings in the baseline (pre-MH onset) and MH (post-MH onset) conditions.

**Patient**	**Condition**	**Morning recordings**	**Evening recordings**	**Average pain level (NRS)**	**Mood (VAS)**
P1	Baseline	4	4	8.67 (±0.20)	4.42 (±0.11)
	MH	4	4	8.50 (±0.16)	4.50 (±0.08)

*For each condition, there were 8 home LFP recordings with corresponding subjective reporting on pain intensity and mood state*.

#### Narrative Description of Patient 2

P2 is a 56-year old man with a history of hypertension and small vessel cerebral ischemic infarct in 2014, attributed to hypertension, resulting in a punctate left thalamic stroke affecting the ventroposterior nucleus. Within days he experienced an onset of residual right hemibody pain, described as burning, aching, pins and needles to the right side of the face, right arm, and right leg, with significant hyperalgesia and allodynia in the right leg. His daily pain, rated a 5 out of 10 at baseline, is exacerbated with activity to 8–10 out of 10. In attempt to mitigate his pain, the patient has reduced his activity at work, and attempted numerous treatments, including physical therapy, TENS, opioids, gabapentin, nonsteroidal anti-inflammatory drugs (NSAIDs), and injections, all without benefit.

He had a less formal background in musical education and performance: formal violin lessons starting at the age of 7 through late adolescence. Unlike P1, this patient denies a prior history of musical hallucinations or involuntary musical imagery.

Upon initiating bilateral ACC stimulation at an amplitude of 3 mA, pulse width of 200 us, and frequency of 130 Hz at contacts 2–0+, he experienced transient episodes of new onset musical hallucinatory activity, which he described as a perception of unfamiliar classical music or a marching band playing. These musical hallucinations lasted for periods of minutes, and generally occurred when he had laid down in bed just before sleep, multiple times per week. Such hypnagogic musical hallucinations occurred in the presence of ongoing stimulation of right ACC with above parameters.

P2's pain has continued to be 4–8/10, with burning, paresthesias, and stabbing pain along the entire left hemibody below the neck, but more pronounced over the left arm and leg. He has difficulty walking for more than 1 mile, but continues to work part-time in the healthcare setting. His main medications include duloxetine and gabapentin, which have been unchanged throughout from the time he was enrolled in the study to the writing of this manuscript.

### Stimulation Related to Musical Hallucinations Activated ACC

**Figure 2:**
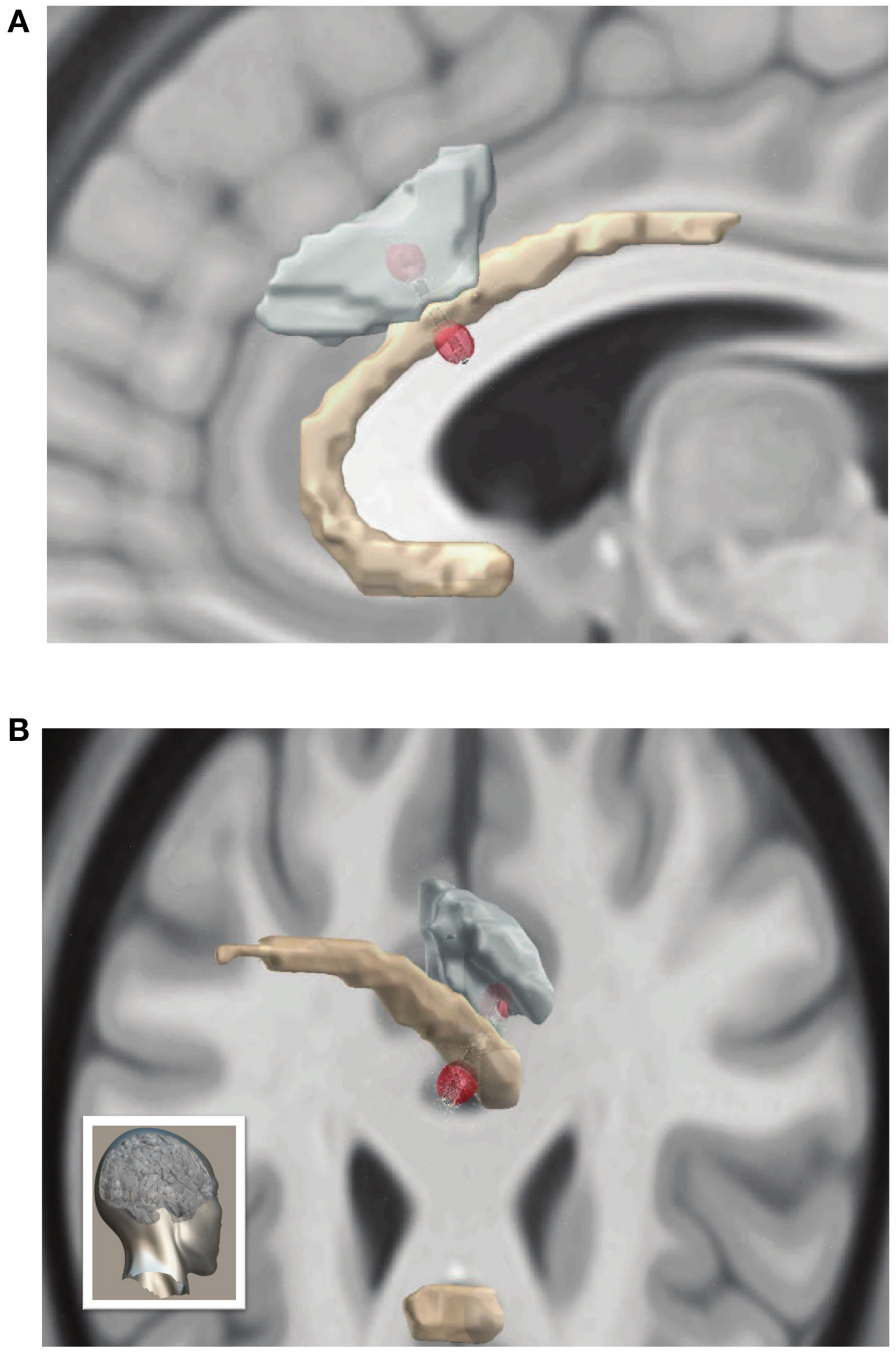
Electrode localization and volume of tissue activated during ACC stimulation **(A)** Sagittal and **(B)** posterolateral (orientation shown in bottom left) views of three-dimensional reconstruction of DBS leads and volume of activated tissue with the MNI brain displayed in the background. Brodmann area 24 in yellow, and Brodmann area 32 in teal. Red spheres represent ends of current dipole, and small white arrows indicate electrical current density.

To reconstruct exact neuroanatomical locations of DBS electrodes, and model the biophysical effects of stimulation we combined preoperative MRI images with postoperative CT scans using the LeadDBS toolkit (43). Electrode tip and contacts were confirmed to reside in the intended target of the rostral ACC, within Broadmann's area 32 just abutting the corpus callosum (Figure 2A). Using the contact locations and stimulation parameters that were first associated with reemergence of MH in P1, we then sought to model the impact of stimulation. Electrical field modeling of stimulation just preceding onset of musical hallucinations in P1 at 6 mA revealed that the volume of tissue activated (VTA) overlapped with Brodmann area 24 by 1.1 mm^∧^3 and Brodmann area 32 by 3.6 mm^∧^3 (Figure 2B). This region encompassed both grey matter of the two inferior most gyri as well as the location of the cingulum bundle. Therefore, stimulation associated with MH in P1 produced an electrical field crossing both grey and white matter structures, likely resulting in both local and remote network effects.

### ACC Alpha and Gamma Power Distinguish MH From Baseline and From Music Listening in N-of-1 Study (P1)

**Figure 3:**
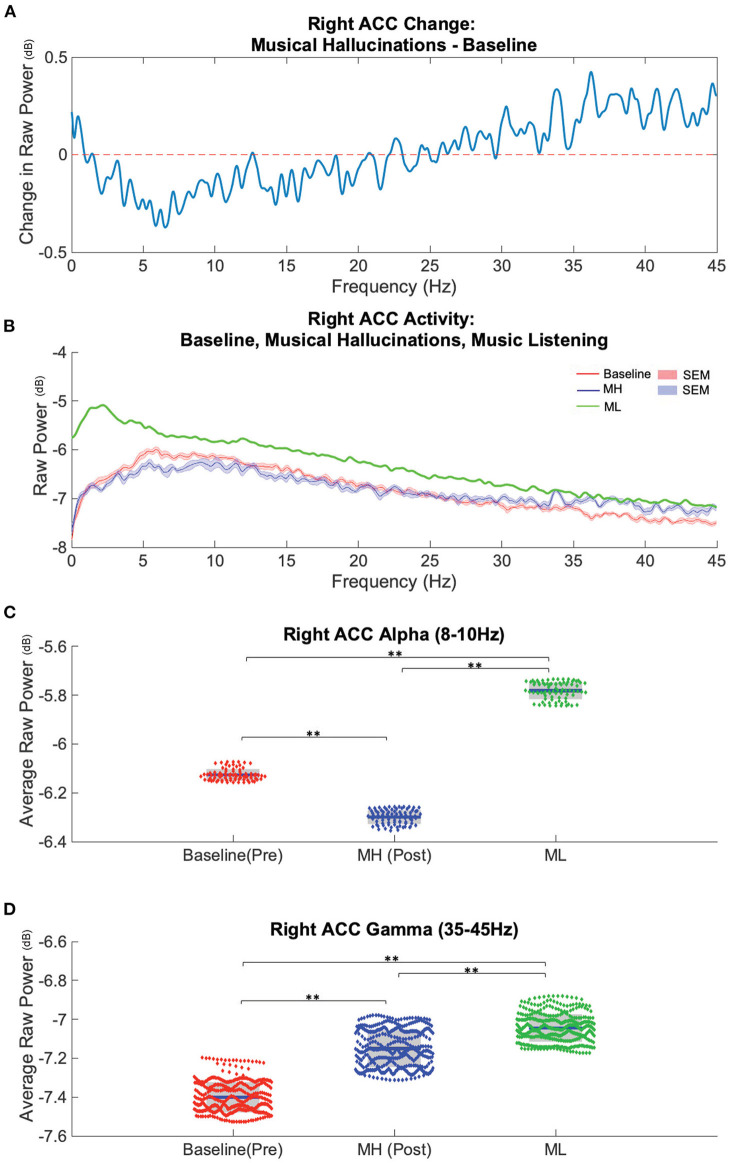
Spectral power in Right ACC distinguishes baseline (pre-MH), musical hallucination (MH), and music listening (ML) conditions. **(A)** Average difference between baseline power (*n* = 8 recordings) subtracted from the post-MH onset (*n* = 8) conditions across analyzed frequencies (0–45 Hz) in ACC. **(B)** Average power spectra and SEM during baseline (red), MH (blue) and music listening conditions (*n* = 1) (green). **(C)** Alpha band power, (8–10 Hz), was lower during MH and higher during ML compared to baseline (blue bar = mean, grey boxes = 95% confidence interval). **(D)** Gamma band power, (35–45 Hz) was higher during MH and ML compared to baseline. **significant difference (*p* < 0.0001).

To assess neural correlates of MH, we analyzed neural recordings from one patient (P1) during three conditions: baseline (muted or no perceptible MH), MH (continuous internal perception of music), and during a separate music listening (ML) task performed in the presence of ongoing MH after reemergence. Power spectra obtained from LFP distinguished these three conditions in the alpha and gamma band (Figure 3). Overall, compared to baseline recordings (no MH ongoing), MH was associated with a decrease in lower frequency power and an increase in higher frequency power (Figures 3A,B) with the spectral curves crossing one another between 25 and 30 Hz. To better characterize changes in power associated with musical hallucinations and music listening, we focused our attention on specific frequency bands of interest: alpha (8–10 Hz) and low gamma (35–45 Hz) across conditions. Compared to baseline (−6.13 ± 0.01), alpha power was significantly decreased during MH (−6.29 ± 0.01, *t-stat* = −42.1, *p* < 0.0001), while ML was associated with an increase in alpha power (−5.78 ± 0.01, *t-stat* = 74.2, *p* < 0.0001). Alpha power during ML was significantly greater than MH (*t-stat* = 104.1, *p* < 0.0001).

Low gamma power showed significant increases for both MH (−7.15 ± 0.01, *t-stat* = 40.9, *p* < 0.0001) and ML (−7.04 ± 0.01, *t-stat* = 68.4, *p* < 0.0001) compared to baseline (−7.40 ± 0.01) (Figure 3C). ML also showed significantly higher low gamma power compared to MH (*t-stat* = 17.9, *p* < 0.0001) (Figure 3C).

## Discussion

We describe induced musical hallucinations associated with electrical stimulation of the rostral anterior cingulate cortex in two patients with refractory chronic neuropathic pain. In one patient who was a musical professional, these musical perceptions reflected a reemergence of previously quiescent internal musical imagery, while in the other patient the phenomenon was associated with hypnagogic states just before sleep. In our N-of-1 analysis, we found that musical hallucinations were associated with decreased alpha power but increased low gamma power compared to baseline. A task involving listening to similar music as that comprising the continuous hallucinations was associated with increased power both in the alpha and low gamma bands compared to both baseline and the MH condition. These data suggest that ACC is differentially involved in the flow of information that underlies internal vs. externally generated auditory percepts. As there is little precedence for studying the neural correlates of musical hallucinations in the context of chronic pain, these findings extend the growing body of literature related to the cerebral localization of chronic pain, hallucinations, and other internally generated percepts towards increased understanding of the role of ACC beyond affective processing.

### A Continuum From Involuntary Musical Imagery to Musical Hallucinations

Complex phantom auditory perceptions are inconsistently defined and often described synonymously with other phantom percepts. For example, while MH are broadly defined as auditory hallucinations in the absence of an external source (48, 49), the Diagnostic and Statistical Manual of Mental Disorders, 5th Edition (DSM−5) defines hallucinations in general as “perception-like experiences that occur without an external stimulus. They are vivid and clear, with the full force and impact of normal perceptions, and not under voluntary control” [(50), page 87]. Additionally, the boundary between voluntary and involuntary musical imagery (INMI), earworms, and musical hallucinations (MH) is often blurred (48). Hemming, for example, defines five conditions of musical imagery: voluntary musical imagery, involuntary musical imagery, permanent involuntary musical imagery, musical hallucinosis, and musical hallucinations (51). While the latter four are involuntary, only MH are described by patients as attributed to an external source (47) and considered pathological.

MH are often described as a more extreme, persistent, or distressing variation of “earworms,” while INMI, coined by Oliver Sacks (52), are described as an everyday, non-pathological and present in most individuals. Sacks proposed that INMI have a basis in long-term memory and reflect involuntary retrieval of stored musical memories. In rare cases, this can be persistent, termed PINMI (51), and occurs in the absence of any identifiable etiology. In this context, it may be considered more specifically that P1 and P2 experienced persistent INMI (PINMI) and INMI, respectively, though we have used the term hallucination throughout for general reader understanding.

Music hallucinations can also be understood in comparison to music listening, in which auditory input is processed in three stages: perception of individual sounds, perception or imagery of pattern in segmented sound, and encoding or recognition of patterned segmented sound (53). For instance, MH have been attributed to abnormal spontaneous activity in auditory regions related to typical musical imagery, which can further be described in terms of thalamocortical dysrhythmia, or limbic-cortical network dysfunction linked to many neurological diseases.

### Chronic Pain and Hallucinations as “Filling in” States

Patient P1 described a “reawakening” of musical hallucinations that were previously muted when her phantom pain became severely intense. This patient effectively has two simultaneous “phantom” percepts: one related to her ongoing phantom limb pain, and another related to the phantom musical perception. One explanation for this “unmasking” of prior hallucinations is that cortical plasticity related to the onset of severe pain somehow “masked” prior internal auditory perceptions, which remained in a latent and barely perceptible state until she received right ACC stimulation. The stimulation may have then selectively modified local and remote circuits associated with this internal representation without necessarily influencing pain perception, suggesting that these circuits are dissociable.

Phantom perception, the conscious awareness of perception despite lack of an external stimulus (54), is a common theme in auditory hallucinations as well as tinnitus and phantom limb pain. In this framework, chronic pain, wherein pain persists for >3 months and often occurs in the absence of identifiable underlying pathology (1), may be considered as a phantom perception. From a Bayesian perspective in which the brain generates predictions about internal and external stimuli (55), chronic pain may be mechanistically similar to hallucinations or tinnitus, wherein an internal subjective state (i.e., pain) is produced as a result of a prediction error between expectation (e.g., normal sensory processing), and reality (e.g., aberrant sensory inputs) (56). Therefore, just as ACC activity may underlie hallucinations reflecting an epiphenomenon of “filling in” of sound in states of impaired hearing, the chronic pain state may represent a similar “filling in” of an aversive subjective experience in response to aberrant peripheral sensory inputs.

### Thalamocortical Dysrhythmia

Thalamocortical dysrhythmia (TCD) has been proposed as a pathological neurophysiological state that underlies a myriad of neurological disorders such as movement disorders, epilepsy, depression, tinnitus, and neuropathic pain (57). In order to define a neural signature of phantom percept and differentiate the underlying electrophysiological activity of simple and complex auditory phantom perceptions found in tinnitus and musical hallucinations, respectively, Vanneste et al. (58) performed source localized EEG studies on these phenomena in healthy chronic hallucinators. This study tested the theory of auditory deafferentation commonly described in these phantom auditory percepts, in which oscillatory alpha activity decreases and theta band activity increases, resulting in surrounding gamma activity known as the “edge effect” (57, 58). The findings suggest that while tinnitus and musical hallucinations share a common neural substrate defined by thalamocortical dysrhythmia, including increased beta activity in the ACC, the complex phantoms of musical hallucinations are associated with activation of brain regions linked to music and language processing. In this case, TCD is defined as abnormally persistent coupled theta-gamma dysrhythmia relayed to the auditory cortex in deafferented thalamic nuclei, binding auditory events into one phantom auditory percept (58). Both neuropathic pain and tinnitus may occur in the deafferented thalamic nuclei and exhibit similar characteristics of TCD. In the study of chronic pain states, TCD has been described to play a potential role in both centrally and peripherally mediated (or neurogenic) neuropathic pain (59, 60). Notably, symptoms of both tinnitus and neuropathic pain can be modified by electrical stimulation of their respective sensory cortices (54). Further, insight on neurological diseases associated with TCD, such as tinnitus and neuropathic pain, could be gained by studying long distance network communications using a combination of electrophysiology and brain connectivity analyses.

### Mismatch Negativity and Neural Pathophysiological Etiologies: Disruption of Connectivity in Temporal and Frontal Cortices

Though there is limited research on musical hallucinations in the presence of chronic pain, controlled studies on the structural, functional, and electrical properties underlying musical hallucinations on other populations have been done. Prior studies have found that the right anterior cingulate is activated in hallucinators while listening to an auditory stimulus and when these subjects had involuntary hallucinations of that same stimulus (61, 62). However, this activation did not occur when subjects voluntarily imagined hearing it, which is referred to as musical imagery or “pseudohallucinations” (48, 63). In an fMRI study on “healthy hallucinators” for instance, Szechtman et al. (62) argue that the ACC (Brodmann area 32) plays a role in “tagging” an auditory event as externally derived. This creates a mismatch in externally directed attention and internal events, a phenomenon described as auditory mismatch negativity (MMN). MMN, a mismatch between sensory memory input and a memory trace of frequent auditory stimuli, has been studied in event related potential (ERP) neuroimaging models to aid the understanding of limbic-cortical structural network dysfunction in schizophrenia patients (64). Several neuroimaging studies have implicated the ACC as one the sources of MMN, though Kirino et al. (64) was the first to apply simultaneous fMRI, EEG, and DTI methods. This multi-faceted approach provides insight into both functional and anatomical discrepancies of the ACC, including a role for the ACC in modulating feed-back/feed-forward deficits between the prefrontal cortex and superior temporal gyrus. While MMN is commonly described in patients with schizophrenia and could reflect possible impaired automatic stimulus discrimination, this concept could serve as a potential framework for understanding the presence of musical hallucination after ACC stimulation.

### Subjective Percepts, ACC, and Auditory Attention

The anterior cingulate has been linked to musical hallucinations for its myriad roles in auditory attention and comprehension (65), the salience network (66), and music and language processing (58). Sadaghiani and colleagues performed fMRI auditory detection tasks and found an association between early sensory cortical activity and activity in the anterior cingulate, anterior insula, and thalamic nuclei; they described these regions as part of an attentional network involving the intrinsic alertness system and associated with task-oriented behavior (65). Additional fMRI studies with auditory tasks have similarly identified the anterior cingulate, insula and medial frontal cortex, in a network supporting auditory attention (67). Studies of auditory attention report increased ACC activity in non-hallucinators during real auditory stimuli compared to hallucinators (65). This suggests a different activation pattern of the ACC and associated brain regions during hallucinated vs. externally heard music.

**Figure 4:**
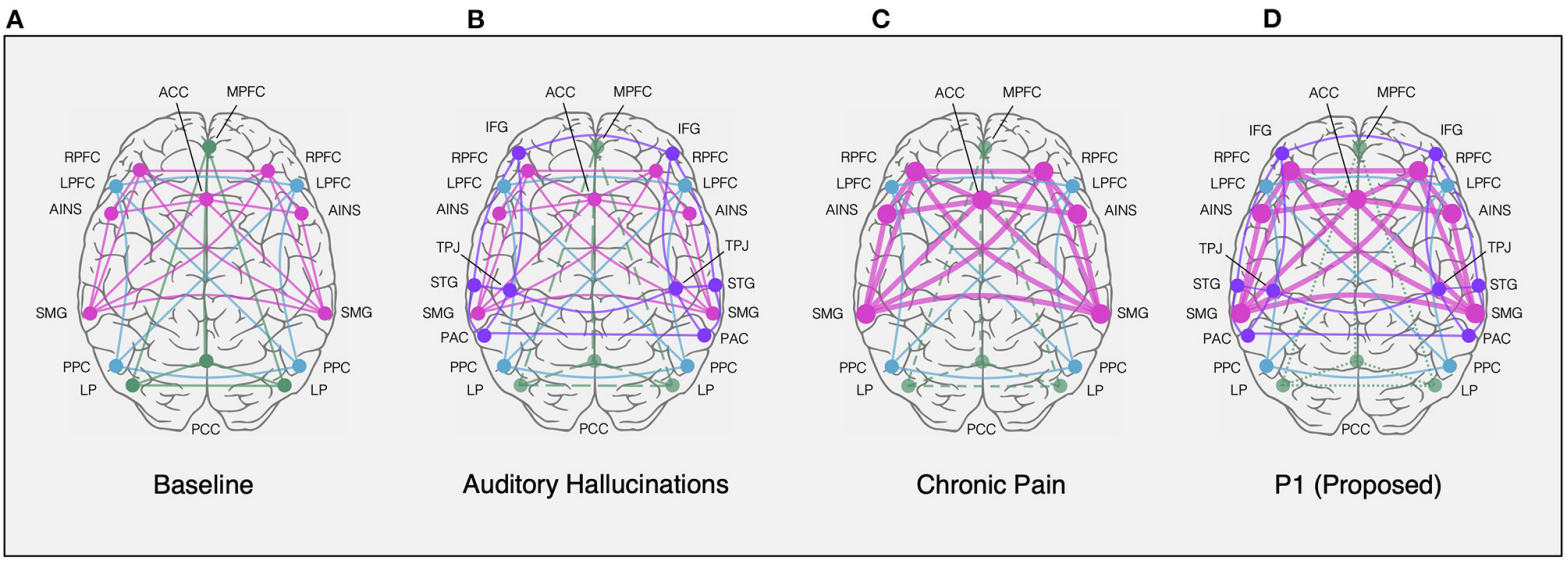
Resting-state network nodes (circles) and connections (lines) depicting the salience network (SN, *pink*), default mode network (DMN, *green*), central executive network (CEN, *blue*), and activated sensorimotor areas (*purple*). Networks are superimposed on axial view of cortical surface from above (anterior towards the top). **(A)** Normal resting connectivity represented in healthy controls (73, 74). **(B)** Resting state activation of sensorimotor areas, including IFG, STG, PAC, and TPJ, in response to DMN withdrawal (i.e., decrease, dashed lines) in auditory hallucinators (75–78). **(C)** Resting state increase in SN connectivity and decrease in DMN connectivity among chronic pain patients (79, 80). **(D)** Proposed combination of chronic pain and hallucinatory network states in response to ACC stimulation demonstrated by DMN withdrawal, sensorimotor shift, and SN hyperactivation, based on previous models (74–77, 79). ACC, anterior cingulate cortex; AINS, anterior insular cortex; IFG, inferior frontal gyrus; LP, lateral parietal cortex; LPFC, lateral prefrontal cortex; MPFC, medial prefrontal cortex; PAC, primary auditory cortex; PCC, posterior parietal cortex; PPC, precuneus cortex; RPFC, rostral prefrontal cortex; SMG, supramarginal gyrus; STG, superior temporal gyrus; TPJ, temporoparietal junction.

The ACC has been described as a critical signal source not only in attention (68, 69), but control of baseline activity in sensory cortices (70). This has been evidenced by coactivation of ACC and auditory cortex during silence seen with positron emission tomography (71) and concordant ACC activation during auditory hallucinations (72). Further, in an fMRI study on auditory cortex activity during silence in healthy participants, Hunter et al. found that increased ACC activation was correlated with co-activation of the auditory cortex and concluded that endogenous auditory activity, such as musical hallucinations, is modulated by the brain's “default mode” comprised of the ACC (37). Studies have further described this altered activation in hallucinators as a shift in resting-state activity of the default mode network to sensorimotor areas, such as the primary auditory cortex, as shown in Figure 4, and other studies (73, 75, 76, 79). Thus, this suggests that attentional biases, such as those that may be triggered by ACC stimulation, can alter the resting state of sensory networks during silence, thereby initiating spontaneous activations that produce hallucinations or imagined percepts.

As described by Mobascher et al. (81) and Boly et al. (82), activity in the anterior cingulate and insula may signify a lack of habituation to a constant phantom percept, such as musical hallucinations, analogous to what has been described for pain (83). Using continuous electrodermal activity and fMRI blood oxygen level dependent (BOLD) measurements during painful laser stimuli, Mobascher et al. (81) describe activity in these somatosensory brain regions in the study of pain habituation which may underlie clinical phenotypes in chronic pain patients, such as an increase in resting salience network activity (Figure 4C) (79). This shift in network activity demonstrates a role for the cingulate cortex in the perception of both painful stimuli and auditory attention. In the current study, we have described one instance in which a patient's baseline auditory phantoms became muted in the presence of severe chronic pain only to reawaken after stimulation of the right anterior cingulate cortex. We additionally found that in a second patient with chronic pain, bilateral anterior cingulate cortex stimulation appeared to initiate musical hallucinations. Thus, the ACC, described as both a potential DBS target for pain relief and as a locus of increased high frequency activity during musical hallucinations, may play a more general role in gating attention towards internally generated percepts. Through this lens, we may begin to reconcile the seemingly disparate phenomena of chronic pain and musical percepts by viewing a role for ACC circuits in regulating attentional shifting of pain and sensory perception in general.

### Limitations and Further Research

In this study, our neural analysis was limited to an N-of-1 case where data was available. Based on this limited sample size, we propose limitations on the generalization of results. Additionally, in this patient, limited recordings were available in each condition: 8 recordings in the “pre-MH” condition, 8 in the “MH” condition, and a single recording in the “ML” condition. While general trends in the patient's recording activity and behavior, such as pain, mood, and time of day, were similar across recordings, there may be other variables associated with the neural features described such as those related to psychological state. For example, since patient P1 experienced MH during right ACC stimulation subsequent to several months of varying stimulation of the bilateral ACC and OFC, we cannot rule out the possibility of a stimulation induced kindling effect, such as the non-convulsant neuroplasticity model of behavioral sensitization, which is an area of research that warrants further investigation. Further, recordings were captured in the ambulatory setting, an uncontrolled environment within the patient's home. The recordings captured by patient P1 were based upon their availability and response to cued reminders, which may not reflect consistent baseline states in pain, mood, and other undefined neurophysiological states. As a result, these findings may reflect individual-specific activity in a musically trained individual that cannot be translated to broader populations of chronic pain or hallucinations, though general results appear to confirm previous findings. Further, the patient may have individual-specific underlying structural or functional abnormalities that predisposed her to stimulation-induced hallucinatory activity. Therefore, any results must be interpreted cautiously.

The ability to detect changes in neural activity during periods where subjects are experiencing musical hallucinations is an important opportunity to further investigate the effects of various stimulation parameters pain-relevant brain regions. Further studies extending from our preliminary findings could, for example, include neurophysiological recordings in more brain regions. Based on hypothesized models of hallucinations, such as the “two-hit bottom-up top-down” models (76), and their associated brain regions, a multi-site network analysis using stereoelectrocephalography (sEEG), for example, could enable simultaneous LFP recording from all regions of interest. This electrophysiological approach to network mapping would supplement the extensive field of functional brain imaging research.

Analysis of LFP changes in response to stimulation is also dependent on the sensing capabilities of the DBS device. In this case, the recordings collected during right ACC stimulation were excluded due to stimulation artifact. Further, neural recordings from the left ACC were excluded from the results due to confounding ECG artifact in the power bands of interest, as previously (84–86). In these studies, authors report on the current leakage of cardiac activity into the IPG due to the proximity of the left pectoral implant to the heart and recommend right IPG sensing for more cleaner recordings (at 3.2× less contamination than left pectoral implants) (84, 85).

This study provides rare evidence of circuit specific changes in the ACC following periods of localized stimulation associated with hallucinations. There are limited cases in which subjects experience a well-defined hallucinatory event in the context of chronic pain. Investigation of these phenomena may provide broader insight into network level communication between brain regions supporting perception of internally generated states.

## Data Sharing

Deidentified individual participant data that underlie the results reported in this article and a data dictionary will be shared using the NIH Brain initiative data sharing platform within 6 months of publication. Data will be shared indefinitely to users who register on the NIH Brain initiative data sharing website: (https://braininitiative.nih.gov/funded-awards/data-archive-brain-initiative%C2%AE-dabi). Shared data can be used to achieve the aims identified in this manuscript. Data sets will include raw neurophysiology data including metadata and MATLAB analytical software code used to generate the main results.

## Data Availability Statement

The raw data supporting the conclusions of this article will be made available by the authors, without undue reservation.

## Ethics Statement

The studies involving human participants were reviewed and approved by Human Research Protection Program (HRPP) at UCSF. The patients/participants provided their written informed consent to participate in this study. Written informed consent was obtained from the individual(s) for the publication of any potentially identifiable images or data included in this article.

## Author Contributions

AS and PSh conceptualized and designed the study and analyzed the data. JS analyzed the electrical field modeling data. AS wrote the manuscript draft. PSt, EC, and PSh also supervised the data collection and writing. All authors assisted with interpretation of the data and contributed writing.

## Conflict of Interest

The authors declare that the research was conducted in the absence of any commercial or financial relationships that could be construed as a potential conflict of interest.
